# Metabolic Flexibility of *Yarrowia lipolytica* Growing on Glycerol

**DOI:** 10.3389/fmicb.2017.00049

**Published:** 2017-01-24

**Authors:** Michael Egermeier, Hannes Russmayer, Michael Sauer, Hans Marx

**Affiliations:** ^1^Christian Doppler-Laboratory for Biotechnology of Glycerol, Department of Biotechnology, BOKU-Vienna Institute of Bio Technology (VIBT), University of Natural Resources and Life SciencesVienna, Austria; ^2^Department of Biotechnology, BOKU-Vienna Institute of Bio Technology (VIBT), University of Natural Resources and Life SciencesVienna, Austria; ^3^ACIB GmbH, Austrian Centre of Industrial BiotechnologyVienna, Austria

**Keywords:** glycerol metabolism, sugar alcohols, citric acid, environmental strain isolation, bioreactor cultivation, dairy derived yeasts

## Abstract

The yeast *Yarrowia lipolytica* is a fascinating microorganism with an amazing metabolic flexibility. This yeast grows very well on a wide variety of carbon sources from alkanes over lipids, to sugars and glycerol. *Y. lipolytica* accumulates a wide array of industrially relevant metabolites. It is very tolerant to many environmental factors, above all the pH value. It grows perfectly well over a wide pH range, but it has been described, that the pH has a decisive influence on the metabolite pattern accumulated by this yeast. Here, we set out to characterize the metabolism of different *Y. lipolytica* strains, isolated from various environments, growing on glycerol at different pH values. The conditions applied for strain characterization are of utmost importance. Shake flask cultures lead to very different results, when compared to controlled conditions in bioreactors regarding pH and aeration. Only one of the tested strains was able to accumulate high amounts of citric acid in shake flask experiments, whereas a group of six strains turned out to accumulate citric acid efficiently under controlled conditions. The present study shows that strains isolated from dairy products predominantly accumulate sugar alcohols at any given pH, when grown on glycerol under nitrogen-limitation.

## Introduction

The yeast *Yarrowia lipolytica* has been studied and investigated for biotechnological applications since the 1960s, when high yield citric acid production in *Y. lipolytica* reached about twice the yield obtained in *Aspergillus niger* (Kurtzman, 2011). Since then, scientific focus concentrated not only on the production of metabolites (Coelho et al., 2010; Barth, 2013), but also on lipid production for biofuel applications (Beopoulos et al., 2008, 2009; Tai and Stephanopoulos, 2013; Rakicka et al., 2015), the production and secretion of heterologous proteins (Nicaud et al., 2002) and on the genetic mechanisms of the dimorphic growth in yeast (Herrero et al., 1999; Ruiz-Herrera and Sentandreu, 2002). However, also from a microbiological point of view the diversity and metabolic flexibility of this yeast is interesting, as these features distinguish *Y. lipolytica* from many other types of yeast. There are three primary lab strains, including the current type-strain NRRL YB-423 (CBS 6124) which has been isolated in 1945 from a maize-processing plant (USA) and later on examined for the secretion of lipases. Some of the widely used strains for the production of microbial lipids and lipases were derived from strain W29 (CBS 7504)—the second lab strain—which has been isolated from sewage in France. The third important lab-strain was isolated from soil in Germany (H 222) and used for the production of organic acids (Otto et al., 2013).

Among the wide spectrum of carbon sources utilized by *Y. lipolytica*, glycerol represents a cost-attractive option as it is generated in high quantity during biodiesel production (Almeida et al., 2012; Khanna et al., 2012). Various studies showed that crude glycerol is readily used as carbon source by many *Y. lipolytica* strains, accumulating citric acid and microbial lipids (Papanikolaou and Aggelis, 2002; Papanikolaou et al., 2002; Beopoulos et al., 2008; Andre et al., 2009). Other metabolites accumulated by this yeast growing on glycerol, include sugar alcohols (polyols) like mannitol, arabitol and erythritol (Rywińska et al., 2013; Miron et al., 2014; Tomaszewska et al., 2014b). An extensive review about the fermentative capability of this yeast on glycerol and other carbon sources was given by Liu et al. (2015). Studies have shown the dependence of the metabolism on the environment. Especially, varying pH conditions have a major impact on the product spectrum formed during bioconversion processes with this yeast (Tomaszewska et al., 2012, 2014a). Growth of many yeast species is limited to a certain pH optimum, with decreasing growth rates when the pH is not optimal. *Y. lipolytica* is very different in this respect and able to adapt to a wide range of pH conditions, shifting the metabolite pattern without impairing growth or the substrate uptake rate. A feature of particular interest—from a microbiological point of view—is the astounding heterogeneity in isolated wild-type strains. While belonging to the same species, according to the current species definition, the strains have different morphologies and entirely different metabolite patterns, when they grow. The three primary lab-strains, as well as many additional local isolates, all seem to have very different properties, depending on their source of isolation. All of these characteristics make *Y. lipolytica* a fascinating microorganism and potentially industrially useful. At the same time these properties require extensive strain characterizations to reveal the full potential of a particular isolate of this yeast. Large scale strain screening experiments are often performed in shake flasks to cover a wide range of cultivation conditions in a reasonable timeframe. These setups make it, however, difficult to ensure tight control of environmental parameters like the dissolved oxygen content or pH and many questions remain unanswered in regard of true strain properties and behavior. To acquire a profound insight into the cellular and metabolic capabilities of microorganisms, bioreactor cultivations represent the most reliable tool to answer queries which emerged from initial strain characterizations in shake flasks. The aim of this work was to define a set of comparable parameters for the characterization of well-known lab strains as well as new isolates. The generation of a comprehensive data set based on the controlled conditions of bioreactor cultivations regarding pH and aeration, assesses the differences and common lines within the metabolic networks of these microorganisms. Additionally, the future potential of each strain to become industrially relevant for the conversion of glycerol into value-added compounds is defined. This study compares the performance of 20 wild-type isolates of the yeast *Y. lipolytica* grown on glycerol batch cultivations (N-limited) in bioreactors under two different pH conditions (3.5 and 5.5). Among them are three primary lab-strains, as well as 12 novel isolates from dairy products. All strains were screened for their potential to produce citric acid, isocitric acid, mannitol, arabitol, and erythritol in the controlled environment of a bioreactor. As the production of metabolites is heavily dependent on the pH applied, two strains with most distinct product patterns were further investigated in bioreactor cultivations under pH conditions in the range of 2.5–7.5.

## Materials and methods

### Microorganisms and media

All strains of *Y. lipolytica* used in this study are listed and described in Table 1. Cells were maintained at −80°C in YPG culture broth supplemented with additional 20% (w/v) of sterile glycerol. Rich medium (YPG) was used for pre-cultures and contained per liter deionized water: 18 g soy peptone, 9 g yeast extract and 20 g of glycerol adjusted to pH 7.5. For the preparation of agar plates, 20 g^*^L^−1^ of agar-agar was added to the YPG medium. Cultures maintained on agar-plates were kept at 4°C for short periods of time. Batch-Cultivations were performed in defined nitrogen-limited media described by Jost et al. (2014). The composition was slightly modified and contained per liter deionized water: 100 g glycerol, 3.1 g (NH_4_)_2_SO_4_, 1.0 g KH_2_PO_4_, 1.3 g Na_2_HPO_4_ × 2 H_2_O, 1.0 g MgSO_4_ × 7 H_2_O, 0.2 g CaCl_2_ × 2 H_2_O, 0.5 g citric acid, 21 mg FeCl_3_, 1 mg Thiamin-HCl, 0.5 mg H_3_BO_3_, 0.06 mg CuSO_4_ × 5 H_2_O, 0.1 mg KI, 0.45 mg MnSO_4_ × H_2_O, 0.71 mg ZnSO_4_ × 7 H_2_O and 0.23 mg Na_2_MoO_4_ × 2 H_2_O.

**Table 1:** **Twenty strains of the yeast *Yarrowia lipolytica* used in this study**.

**Strain number**	**Isolated from**	**Strain number**	**Isolated from**
CBS 6124^a^	Maize process. plant	HA 827^d^	Cheese
CBS 7504 (W29)^a^	Sewage	HA 828^d^	Cheese
DSM 1345^b^	Petroleum product	HA 829^d^	Cheese
DSM 3286^b^	Unknown	HA 830^d^	Cheese
DSM 21175^b^	Airplane fuel tank	HA 831^d^	Cheese
H222^c^	Soil	HA 832^d^	Cheese
CBS 6114 (HA 991)^d^	Plastics	HA 833^d^	Cheese
CBS 7034 (HA 992)^d^	Soil	HA 834^d^	Cheese
HA 807^d^	Cheese	HA 1251^d^	Butter
HA 826^d^	Cheese	HA 1252^d^	Cheese

a *CBS-KNAW Fungal Biodiversity Center*.

b *Leibniz Institute DSMZ-German Collection of Microorganisms and Cell Cultures*.

c *Dresden University of Technology*.

d *ACBR-Austrian Centre for Biological Resources and Applied Mycology*.

### Shake flask cultivations

Strains were cultivated at 30°C and 180 rpm shaking in 500 mL baffled shake flasks containing 25 mL of 2x YNB medium, containing per liter deionized water: 3.4 g YNB (yeast nitrogen base—Difco™), 50 g pure glycerol and 1.0 g (NH_4_)_2_SO_4_. Initial pH was set to pH 5.5 and cultures were inoculated with an OD_600_ of 0.1. All shake flask experiments were performed in two biological replicates with standard deviations of ≤ 20%.

### Batch-cultivations

All cultivations were carried out in DASGIP bioreactor systems (Eppendorf AG, Hamburg, Germany) with four parallel bioreactors and a maximum working volume of 1200 ml. The reactors were sterilized by autoclaving at 121°C for 20 min, and the culture medium was added by sterile filtration into the reactor. Aerobic conditions with 50% dissolved oxygen were maintained by automatic control of the stirrer speed and inlet air. Temperature was set to 30°C and the pH adjusted by automated addition of 5 M NaOH. Two pH conditions (3.5 and 5.5) were tested for each strain. The antifoam agent used was 5% (w/v) Struktol (SB 2121, Schill+Seilacher, Hamburg, Germany). For pre-cultures, 100 mL of YPG medium was inoculated with cells from a single colony grown on YPG-plates and incubated overnight in a shaker at 30°C and 180 rpm. Cells were harvested in the exponential phase and washed with sterile deionized water. 500 ml of culture medium was then inoculated with an OD_600_ of 1 from the exponentially growing pre-culture. All cultivations had an initial pH of 5.5. For lower pH settings (3.5) pH regulation was activated after a drop of the pH from 5.5 to 3.5 due to acidification of the culture media during growth phase within the initial 8 h. In case of higher pH settings 5 M NaOH was gradually added to the medium to achieve a slow increase in pH within 8 h of cultivation. Production phase was entered not before 12 h of cultivation when nitrogen was depleted in the culture medium. All bioreactor cultivations were performed in two and three biological replicates for strain screening and pH-condition screening, respectively. The standard deviation between bioreactor cultivations did not exceed 15%.

### Analytical procedures (CDW, HPLC, enzymatic assay)

Six milliliter samples were taken at regular intervals throughout the whole cultivation duration. Optical density was determined by appropriate dilution of culture broth to an absorbance of 0.1–0.7 at 600 nm determined with a photometer (Biochrom WPA CO8000 Cell Density Meter). Additionally, cell dry weight (CDW) was determined by desiccation of washed cell pellets obtained from 4 mL culture broth. Samples were centrifuged at 15,000 g for 10 min and dried at 105°C for at least 24 h. The concentrations of residual glycerol, citric acid, mannitol, arabitol, and erythritol in the culture broth were determined by HPLC analysis (Shimadzu, Korneuburg, Austria) using a method for the detection of carboxylic acids, sugars and polyols previously established in our lab (Pflügl et al., 2012). A BIO-RAD Aminex® HPX-87H Column 300 × 7.8 mm (Bio-Rad Laboratories, Inc., USA) and a refraction index detector (RID-10A, Shimadzu, Korneuburg, Austria) were used. The column was operated with 0.004 M H_2_SO_4_ as mobile phase at 60°C temperature, 0.6 ml^*^min^−1^ flow rate. The obtained values detected for citric acid by HPLC measurement also contained isocitric acid as the two substances cannot be separated by the method described above. Therefore, a D-Isocitric Acid Assay Kit from Megazyme (K-ISOC 01/14, Megazyme International, Ireland) was used to determine isocitric acid content with a detection limit of 0.1 g^*^L^−1^ and all samples were measured in duplicates. To obtain the final amount of citric acid in the samples, results for isocitric acid content were subtracted from total citric acid concentrations obtained by HPLC measurement. During the bioreactor cultivations the ammonium concentration in the cultivation medium was determined by a Hach-Lange HQ30d portable meter and an ISENH4181 ammonium selective electrode (Hach Lange GMBH, Düsseldorf, Germany). It is known, that the ammonium measurement is strongly influenced by media components (e.g., ${\text{SO}}_{4}^{2+}$ ions), therefore all obtained values were calculated back to the amount of (NH_4_)_2_SO_4_ used in the media. An initial (NH_4_)_2_SO_4_ concentration of 3.09 g^*^L^−1^ corresponds to 47 mmol^*^L^−1^ of NH^4+^ at the beginning of bioreactor cultivations.

## Results

To ensure comparability and the opportunity for media improvements in the future it was crucial for us to avoid complex media components (e.g., yeast extract) and to use defined media in both shake flask and bioreactor cultivations. Yeast nitrogen base-medium was used in the first part of the study as it is a widely used medium for initial strain screening experiments. For bioreactor cultivations a slightly modified media composition published by Jost et al. (2014) was selected.

### Shake flask cultivations

**Figure 1:**
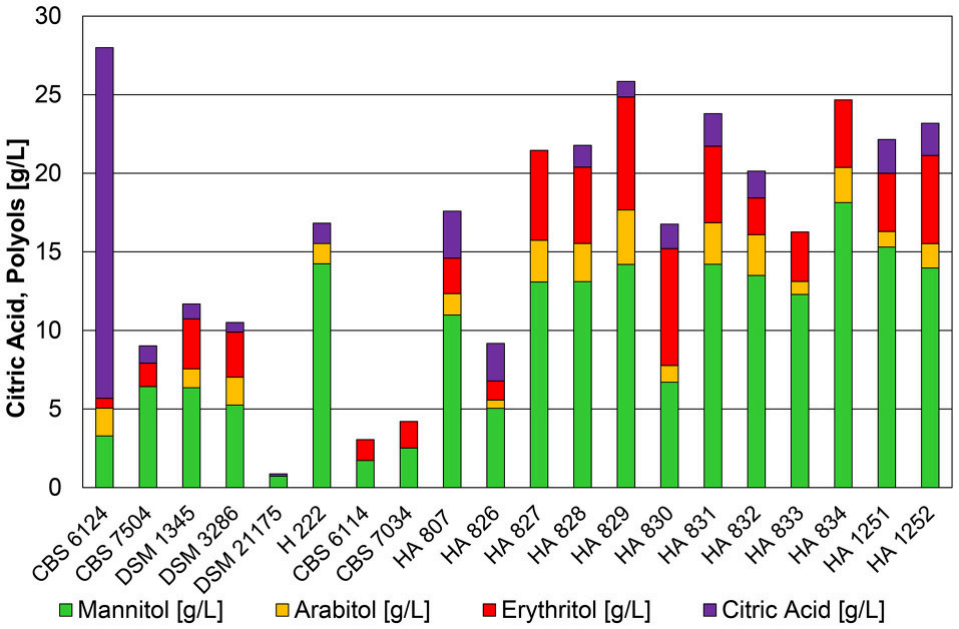
Product pattern of *Y. lipolytica* strains cultivated in 500 mL baffled shake flasks containing 25 mL of 2x YNB medium with 50 g^*^L^−1^ glycerol and 1.0 g^*^L^−1^ (NH_4_)_2_SO_4_ at 30°C on a rotary shaker with 180 rpm after 200 h of cultivation. The initial pH of 5.5 decreased for all cultivations to values between 1.8 and 2.2. The presented data represents the mean of two biological replicates with *SD* ≤ 20%.

Strains of *Y. lipolytica* cultivated in shake flasks on defined YNB medium with 5% pure glycerol grew very slowly and did not exceed 6.8 g^*^L^−1^ of dry biomass after 200 h of cultivation (Table S1). The initial pH of 5.5 decreased due to acidification during growth phase and the formation of citric acid to 1.8–2.2. Figure 1 summarizes the results of shake flask cultivations. The main metabolite produced was mannitol, although some of the strains were not able to convert the entire glycerol even after 200 h of cultivation. The only strain that produced high amounts of citric acid (22.3 g^*^L^−1^, Y_P/S_ = 0.24 g^*^g^−1^) at low pH conditions in shake flask experiments was the type strain CBS 6124. A similar result using the same strain (= NRRL YB-423) was obtained by Levinson et al. (2007) with 21.6 g^*^L^−1^ and a higher yield of 0.54 g^*^g^−1^. Results shown in Figure 1 already indicate a preference for the conversion of glycerol to polyols, especially to mannitol, at low pH in strains isolated from cheese and butter. Strain H222 was the only primary lab-strain producing high amounts of mannitol, whereas strain CBS 7504 (W29) showed the lowest productivity of metabolites on glycerol in the shake flask experiments. This result stands in contrast to shake flask cultivations performed by Papanikolaou et al. (2013) in which CBS 7504 was able to convert 40 g^*^L^−1^ of crude glycerol into 12.5 g^*^L^−1^ of biomass, 19 g^*^L^−1^ of citric acid with no additional formation of polyols within 160 h of cultivation. However, a phosphate buffered medium with a final pH of 4.8–6.0 with additional 2.5 g^*^L^−1^ of yeast extract as nitrogen source were used during these cultivations. The absence of these media components in YNB medium used in the present study, as well as the significantly lower pH of around 2.0 might be an explanation for the reduced ability of CBS 7504 to convert glycerol into biomass, citric acid and polyols.

### Bioreactor cultivations

**Figure 2:**
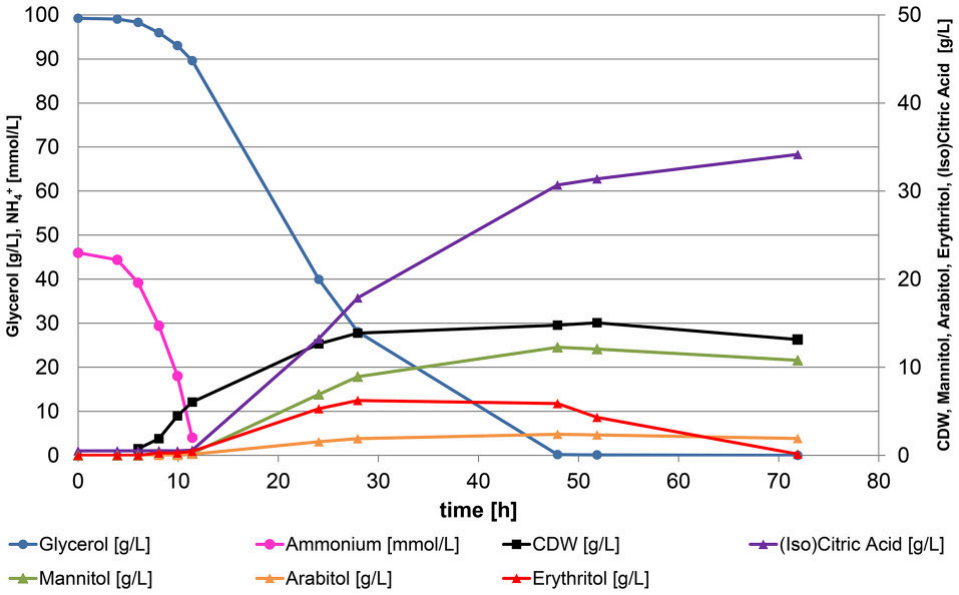
**Representative growth curve for all bioreactor cultivations of *Y. lipolytica***. Strain DSM 3286 cultivated in a bioreactor containing 500 mL of nitrogen-limited media at pH 4.5 with 100 g^*^L^−1^ glycerol as carbon source and 3.1 g^*^L^−1^ of (NH_4_)_2_SO_4_ as sole nitrogen source. Nitrogen-limitation triggers the formation of citric acid and polyols after 12 h, when the entire ammonium from the media is consumed.

The media composition with 10% of pure glycerol for bioreactor screenings was selected after several media recipes published in literature were tested for their performance in DASGIP bioreactors. YNB medium turned out to cause a very long lag phase in bioreactor compared to other media, but a medium derived from Jost et al. (2014) allowed a significant reduction of cultivation time to 72 h. In contrast to the shake flask experiments, all 20 strains showed robust growth on this media in bioreactor cultivations at both tested pH conditions and converted the entire carbon source to biomass, citric acid, isocitric acid, and polyols during production phase upon nitrogen-limitation within 48–72 h of cultivation. In Figure 2 a representative growth curve is shown for all bioreactor cultivations. The entire ammonium provided was consumed in all experiments between 11 and 13 h of cultivation. Further growth of the yeast culture was prohibited in this stage of the cultivation and the cells entered nitrogen-limited cultivation conditions. *Yarrowia lipolytica* is a dimorphic yeast. It can grow as single cells or in filamentous form. The connection between the morphological appearance and productivity is still unclear. Interestingly, at the conditions applied, all tested strains grew mainly in yeast-like form throughout the experiment, except DSM 3286 formed pseudo-mycelia during both cultivation conditions. Final biomass concentrations remained constant after nitrogen depletion between 11 and 24 g^*^L^−1^ of cell dry weight with higher biomass yields for the strains growing in yeast-like form.

### Low pH leads to polyol production

**Figure 3:**
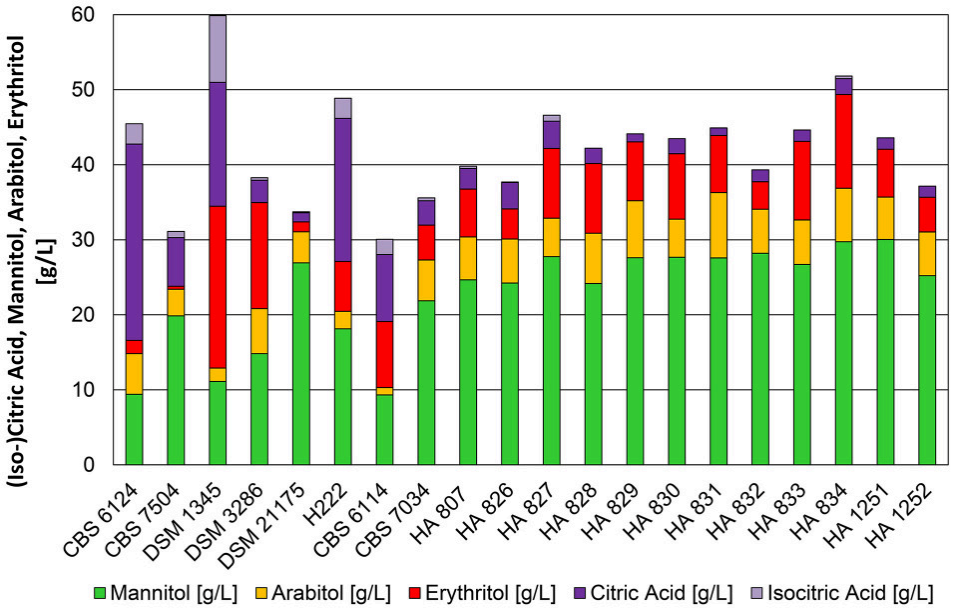
Product pattern of *Y. lipolytica* strains after 48 h of cultivation in bioreactors containing 500 mL of nitrogen-limited media at pH 3.5 with 100 g^*^L^−1^ glycerol as carbon source. Constant dissolved oxygen content of 50% was applied with a temperature of 30°C. The presented data represents the mean of two biological replicates with *SD* ≤ 15%.

At a pH of 3.5 and after 48 h of cultivation 15 out of 20 strains converted glycerol mainly into polyols (Figure 3) with yields of 0.30–0.47 g^*^g^−1^. In accordance to shake flask cultivations the type strain CBS 6124 produced high amounts of citric acid (28.9 g^*^L^−1^). The isocitric acid content was 9.3%. Also strain H 222 secreted more than 20 g^*^L^−1^ of citric acid (isocitric acid content 12%—Table S2) at low pH. Surprisingly, after a marginal product formation in the shake flasks, DSM 1345 converted excess glycerol into citric acid and reached concentrations of 25.4 g^*^L^−1^ with a relatively high isocitric acid content of 33% and simultaneous production of polyols (11 g^*^L^−1^ mannitol, 21.6 g^*^L^−1^ erythritol). These divergent results of the shake flask experiment compared to cultivation in bioreactors underline the importance of media composition and a controlled environment in bioreactor cultivations for comprehensive strain characterization experiments. The sampling point after 48 h of cultivation, when glycerol is already depleted in the culture, represented the peak for the conversion of glycerol to polyols. This holds true especially for strains which are simultaneously producing citric acid. Erythritol, and to a lesser extent also mannitol, were re-consumed by these strains after glycerol depletion to be further converted into citric acid and lead to a decrease in polyol titers. In a study from 2009 Andre et al. hypothesized that re-consumption of secreted metabolites is exclusively used for maintenance requirements. In contrast to this and in accordance to the observations made in the present study, the utilization of erythritol for the production of citric acid has been described earlier this year for *Y. lipolytica* strain LFMB 20 after 140 h of shake flask cultivation on glycerol (Papanikolaou et al., 2016). Therefore, we decided to use this sampling time point for a better visualization of the potential of polyol formation in *Y. lipolytica* strains during bioreactor cultivations.

### Higher pH triggers formation of citric acid

**Figure 4:**
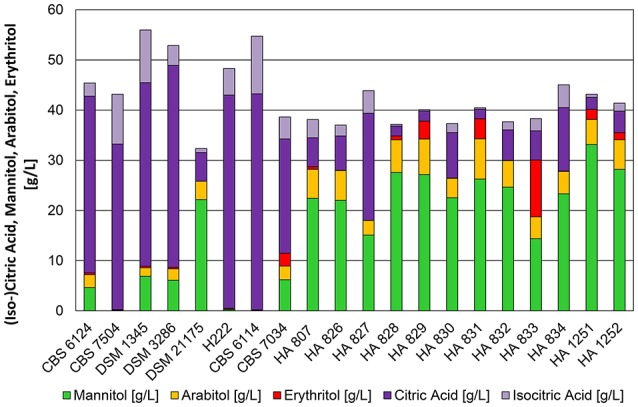
Product pattern of *Y. lipolytica* strains after 72 h of cultivation in bioreactors containing 500 mL of nitrogen-limited media at pH 5.5 with 100 g^*^L^−1^ glycerol as carbon source. Constant dissolved oxygen content of 50% was applied with a temperature of 30°C. The presented data represents the mean of two biological replicates with *SD* ≤ 15%.

The pH optimum for organic acid production in *Y. lipolytica* was around 5.5. Figure 4 displays 20 strains of which a group of 6 strains (CBS 6124, CBS 7504, DSM 1345, DSM 3286, H222, and CBS 6114) produced more than 30 g^*^L^−1^ of citric acid after 72 h of cultivation, whereas the majority of the strains continued producing mannitol as main metabolite. In a recent publication Papanikolaou et al. (2016) demonstrated the strain dependency in mannitol and erythritol production from glycerol even at neutral pH, which is in accordance to the results shown in this study and by Andre et al. (2009). Interestingly, all of the primary lab strains belonged to the citric acid producing strains, whereas strains of *Y. lipolytica* isolated from dairy products predominantly produce polyols. Similar conversion yields for sugar-alcohols (>0.3 g^*^g^−1^) at pH 3.5 and 5.5 were achieved for these strains. In general, the conversion yield was higher for citric acid producing strains and could reach up to 0.5 g^*^g^−1^ of glycerol. Polyols were typically accumulated during the cultivation and partially re-consumed after glycerol depletion to be further converted into citric acid as already observed for cultivation conditions at pH 3.5. Noteworthy is, that three strains completely down-regulated the formation of polyols at the pH of 5.5 (CBS 7504, H222, CBS 6114—Figure 4). These strains did not even accumulate polyols at an earlier stage of the cultivation at a pH of 5.5 (data not shown).

### The influence of pH

To further investigate the influence of pH on the metabolite pattern of *Y. lipolytica*, two strains with distinct product patterns were selected (DSM 3286 and DSM 21175) and characterized in more detail. To our knowledge, DSM 21175 has never been described in literature so far. This strain performed very poorly in shake flasks but showed growth, independent of the environmental pH in the bioreactor accumulating mainly mannitol. DSM 3286 has already been described for the production of lipases from plant oils (Darvishi et al., 2009) and the conversion of glucose to citric acid (Anastassiadis et al., 2002; Mirbagheri et al., 2011). In our experiments this strain produced significant amounts of mannitol and erythritol in shake flask experiments. One interesting feature of this strain is the ability to shift its metabolite pattern in a bioreactor from polyol production at pH 3.5 toward citric acid formation at pH 5.5. Additional bioreactor cultivations with both strains were carried out at pH conditions in a range between 2.5 and 7.5 and all experiments were performed in triplicates.

**Figure 5:**
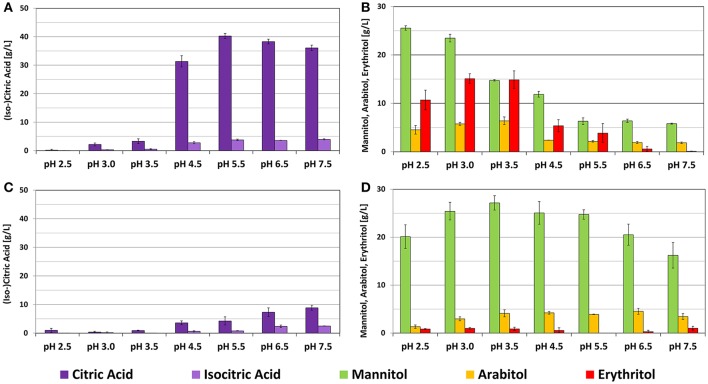
**Results of *Y. lipolytica* strain screening in bioreactor cultivations under different pH conditions. (A)** DSM 3286 after 72 h of cultivation, **(B)** DSM 3286 after 48 h of cultivation, **(C)** DSM 21175 after 72 h of cultivation, **(D)** DSM 21175 after 48 h of cultivation. Both strains have a distinct product pattern: DSM 3286 with pH-dependent production of polyols and citric acid and DSM 21175 mainly producing mannitol. The presented data represents the mean of three biological replicates.

Results summarized in Figure 5 illustrate the dependency of cellular performance of a certain strain isolate on the source of isolation. Importantly, growth behavior remained stable for both strains throughout the whole pH spectrum with slight decrease in growth rates only at the extreme values of the tested pH-range (Table 2). Cultivations of DSM 3286 revealed several pH optima for the production of polyols (low pH) and citric acid (neutral pH). Mannitol was the main polyol produced from glycerol by this strain and the highest titers were achieved at pH 2.5 with a total conversion yield for polyols of >0.4 g^*^g^−1^. At the same time, pH 3.5 seemed to be optimal conditions for the formation of erythritol. Also the production of arabitol reached its peak at pH 3.5. Citric acid was preferably formed at pH values higher than 4.5, with an optimum around pH 5.5 and reached conversion yields of >0.4 g^*^g^−1^ (Table 2). These findings underline the remarkable flexibility of the metabolism of *Y. lipolytica* depending on the environmental conditions.

**Table 2:** **Strain characteristics of *Y. lipolytica* DSM 3286 and DSM 21175 tested under different pH conditions in bioreactor cultivations (n.d.—not detected)**.

**Strain name**	**pH**	**μ _max_ (h^−1^)**	**DCW (g/L)**	**Yield Polyols (g/g)**	**Yield Citric Acid/Isocitric Acid (g/g)**	**Isocitric Acid (%)**
DSM 3286	2.5	0.30 ± 0.05	17.5 ± 1.0	0.41	0.00	n.d.
	3.0	0.35 ± 0.04	14.1 ± 0.9	0.43	0.02	14.0
	3.5	0.29 ± 0.04	14.7 ± 0.9	0.36	0.04	14.1
	4.5	0.35 ± 0.07	11.2 ± 2.6	0.20	0.34	8.2
	5.5	0.31 ± 0.01	11.7 ± 0.7	0.13	0.44	8.6
	6.5	0.35 ± 0.07	10.2 ± 1.8	0.09	0.42	8.6
	7.5	0.34 ± 0.03	14.9 ± 1.8	0.08	0.40	10.0
DSM 21175	2.5	0.43 ± 0.09	20.8 ± 0.4	0.26	0.01	n.d.
	3.0	0.47 ± 0.01	20.9 ± 0.6	0.30	0.00	n.d.
	3.5	0.39 ± 0.04	20.1 ± 2.2	0.29	0.02	15.8
	4.5	0.45 ± 0.14	21.7 ± 0.3	0.30	0.04	17.5
	5.5	0.39 ± 0.05	22.4 ± 3.3	0.29	0.05	15.9
	6.5	0.47 ± 0.03	23.2 ± 1.3	0.26	0.09	24.5
	7.5	0.39 ± 0.07	22.3 ± 2.0	0.22	0.12	21.1

In contrast, the metabolite pattern of DSM 21175 was restricted to the conversion of glycerol to mannitol throughout the whole pH-range tested. The product formation rate of DSM 21175 at pH 2.5 is slower and therefore the product titer after 72 h of cultivation is significantly lower. With prolonged cultivation time, however, also at pH 2.5 concentrations around 25 g^*^L^−1^ of mannitol were achieved (data not shown). Even with an increasing pH toward 7.5 only small amounts of citric acid were formed by DSM 21175. Noteworthy is the significant increase of isocitric acid content under these conditions. Summing up, these results revealed a distinct dependency of metabolite pattern on the strain isolate and emphasized the necessity for strictly controlled cultivation conditions for characterization purposes, which can only be achieved in a bioreactor.

Furthermore we could demonstrate that minimal media composition is sufficient for growth of *Y. lipolytica* on pure glycerol and the bioconversion of excess carbon into industrially relevant metabolites. In Table 3 a comparison between yields and titers from literature is listed. The values for citric acid and mannitol are comparable with cultivations supplemented with yeast extract as complex media compound and are among the highest ever reported in literature.

**Table 3:** **Comparison of *Y. lipolytica* strains screened under different pH conditions in bioreactor batch cultivations for the production of polyols and citric acid on pure glycerol as sole carbon source**.

**Strain name**	**pH**	**CDW (g/L)**	**Mannitol (g/L)**	**Yield Mannitol (g/g)**	**Erythritol (g/L)**	**Yield Erythritol (g/g)**	**Citric Aicd (g/L)**	**Yield Citric Acid (g/g)**	**Isocitric Acid (%)**	**References**	**Nitrogen Source**
*Y. lipolytica* A UV'1	3.00	–	27.6	0.16	59.3	0.35	–	–	–	Tomaszewska et al., 2012	2 g^*^L^−1^ NH_4_Cl + 1 g^*^L^−1^ yeast extract
*Y. lipolytica* A-15	3.00	–	23.0	0.16	28.0	0.19	–	–	–	Tomaszewska et al., 2012	2 g^*^L^−1^ NH_4_Cl + 1 g^*^L^−1^ yeast extract
*Y. lipolytica* Wratislavia K1	3.00	–	12.8	0.12	42.0	0.22	–	–	–	Tomaszewska et al., 2012	2 g^*^L^−1^ NH_4_Cl + 1 g^*^L^−1^ yeast extract
	3.00	19.3	15.1	–	40.7	0.28	2.6	–	–	Tomaszewska et al., 2014a	3 g^*^L^−1^ (NH_4_)_2_SO_4_ + 1 g^*^L^−1^ yeast extract
	4.50	23.7	6.1	–	32.2	0.22	45.9	0.31	–	Tomaszewska et al., 2014a	
	5.50	17.3	6.3	–	26.5	0.18	65.0	0.43	5.8	Tomaszewska et al., 2014a	
	5.50	15.7	8.3	–	30.2	–	53.3	0.34	4.8	Rywińska et al., 2010	3 g^*^L^−1^ NH_4_Cl + 1 g^*^L^−1^ yeast extract
*Y. lipolytica* A-101	5.50	17.1	4.9	–	n.d.^#^	–	66.5	0.44	21.1	Rywińska et al., 2010	3 g^*^L^−1^ NH_4_Cl + 1 g^*^L^−1^ yeast extract
*Y. lipolytica* DSM 3286	3.00	14.1	23.5	0.23	15.1	0.15	2.2	0.02	13.3	This study^**^	3.1 g^*^L^−1^ (NH_4_)_2_SO_4_
	4.50	11.2	12.3	0.12	5.9	0.05	34.1	0.34	8.2		
	5.50	11.7	6.3	0.06	3.9	0.04	44.0	0.44	8.6		
*Y. lipolytica* DSM 21175	3.00	20.9	25.4	0.27	1.0	0.01	0.6	0.01	n.d.	This study^**^	3.1 g^*^L^−1^ (NH_4_)_2_SO_4_
	4.50	21.7	25.1	0.26	0.6	0.01	4.2	0.04	13.1		
	5.50	22.4	24.7	0.25	n.d.	–	5.1	0.05	14.9		

# *n.d.—not detected*.

* *Erythritol has been reconsumed after glycerol depletion*.

** *Maximum values produced after 48 and 72 h for polyols and citric acid, respectively*.

## Discussion

The characterization of wild-type isolates represents the first step in understanding the physiology and the underlying regulatory mechanisms of novel organisms and a potential microbial cell factory. In this work we have shown, that the environmental conditions and the source of strain isolation play a crucial role in the generation of comprehensive and comparable datasets for the characterization of wild-type strains. Small scale experiments in shake flasks provide a fast and inexpensive method to get a first glance on the cellular characteristics of a novel strain. One of the widely used media recipes for shake flask screenings is YNB with an additional nitrogen source. The pH conditions are either controlled by buffering agents (e.g., phosphate salts) or the manual addition of alkaline reagents (e.g., KOH). Both methods often lead to a fluctuating pH environment during the cultivation and consequently often to diverging results. Moreover, the addition of salts can have a decisive impact on strain performance (Tomaszewska et al., 2014a). Therefore, we decided to use unbuffered YNB medium in shake flask experiments, which lead to a decrease in pH from 5.5 to values around 2.0. Indeed, the three primary lab strains used in academia over the past decades show different capabilities to metabolize excess carbon under these conditions. While CBS 6124 converted glycerol to significant amounts of citric acid CBS 7504 formed only small amounts of total polyols (7.9 g^*^L^−1^) and H222 secreted 14.2 g^*^L^−1^ of mannitol. However, cultivated on minimal media in bioreactors, all three strains showed robust growth and the ability to form citric acid under nitrogen-limited conditions. This leads us to the conclusion, that only the controlled environment of a bioreactor (especially regarding pH) reveals the full potential of a microorganism to convert a given substrate into microbial metabolites of industrial significance.

One of the abundant substrates for microbial conversion is glycerol derived from biodiesel production (Almeida et al., 2012). In this regard, 20 strains of *Y. lipolytica* have been characterized in bioreactors for their ability to convert (pure) glycerol into polyols and citric acid under two different pH conditions. Optimal growth is for many species limited to a quite narrow range of pH, temperature, aeration and nutrients. However, *Yarrowia lipolytica* is different. It possesses the ability to adapt to an astonishingly wide range of for example pH conditions without any significant effects on growth behavior or substrate uptake rate. Concurrently, for some strains of *Y. lipolytica* the adaption to changes in extracellular pH conditions leads to significant alterations in the metabolite pattern produced. Strikingly, different strains of *Y. lipolytica* metabolize glycerol entirely different. While all of the strains belong to the same species according to the currently accepted species definition, they seem to be very different. For example, *Yarrowia lipolytica* CBS 6114 produced no polyols at all but 43.1 g^*^L^−1^ of citric acid and 11.5 g^*^L^−1^ of isocitric acid. On the other hand, at the same cultivation conditions *Y. lipolytica* HA 829 converted glycerol to 27.2 g^*^L^−1^ of mannitol, 7.1 g^*^L^−1^ of arabitol and 3.6 g^*^L^−1^ of erythritol with negligible amounts of citric acid (Table S3). Just observing the metabolic activity, one would not assume that all of the organisms we looked at are of the same species. However, it becomes clear that strains isolated from similar origins behave similar. Strains isolated from dairy products predominantly form sugar alcohols with glycerol as sole carbon source - independent from the environmental pH. Other strains switch to citric acid formation at neutral pH. A recent study by Rakicka et al. (2016) investigated the potential of several yeast species from the *Yarrowia* clade to utilize glycerol and other carbon sources in media with different salt concentrations and pH conditions. According to their results the performance of *Y. lipolytica* to produce citric acid is unchallenged within this genus. Regarding the formation of polyols, however, other species turned out to be even more efficient. Especially *Yarrowia divulgata* (mannitol: 28 g^*^L^−1^, Y_P/S_ = 0.25 g^*^g^−1^, erythritol: 35.4 g^*^L^−1^ Y_P/S_ = 0.32 g^*^g^−1^), *Candida hollandica* (mannitol: 34.2 g^*^L^−1^, Y_P/S_ = 0.31 g^*^g^−1^, erythritol: 33.4 g^*^L^−1^ Y_P/S_ = 0.30 g^*^g^−1^) and *Candida oslonensis* (mannitol: 34.3 g^*^L^−1^, Y_P/S_ = 0.29 g^*^g^−1^, erythritol: 44.6 g^*^L^−1^ Y_P/S_ = 0.40 g^*^g^−1^) turned out to be good natural producers of polyols and may also serve as gene donors for engineered strains of *Y. lipolytica* (Rakicka et al., 2016). Nevertheless, results obtained in our work indicate, that strains of *Y. lipolytica* isolated from dairy products might be able to close the gap and compete with their relatives from the *Yarrowia* clade. These strains lack the ability to produce citric acid at neutral pH conditions. Instead they reach titers of up to 29.7 g^*^L^−1^ and 12.5 g^*^L^−1^ for mannitol and erythritol respectively (see strain HA 834, Table S2) with a total conversion yield for polyols of up to 0.47 g^*^g^−1^ from pure glycerol at low pH. As we could show for *Y. lipolytica* DSM 3286, the adjustment of process conditions (pH), as well as the salt concentration in the media (demonstraded by Tomaszewska et al., 2014a), can shift the metabolite pattern toward a certain direction (e.g., from mannitol to erythritol formation). According to Fleet (1990) some of the common properties of yeast species isolated from dairy products is their ability to assimilate citric acid and withstand low pH conditions. Understandably enough that the cultivation of *Y. lipolytica* strains isolated from dairy products tend to accumulate polyols instead of citric acid. It will be interesting to see, if the differences between the strains are genetically manifest, or if other, for example epigenetic, regulations dictate the metabolism in this case.

## Author contributions

ME and HR designed and performed the experiments. They contributed to analysis and interpretation of the data. HM and MS analyzed and interpreted the data, and drafted the manuscript. All authors read and approved the final manuscript.

### Conflict of interest statement

The authors declare that the research was conducted in the absence of any commercial or financial relationships that could be construed as a potential conflict of interest.
