# Supplementary material for: Metabolic Flexibility of Yarrowia lipolytica Growing on Glycerol
Source: Front Microbiol. 2017 Jan 24;8:49. doi: 10.3389/fmicb.2017.00049 (PMC5258708; doi:10.3389/fmicb.2017.00049)
Supplement: Supplementary file 1 [file Table1.DOCX]

**Table S1 – Strain screening results for twenty *Y. lipolytica* strains cultivated in shake flasks with an initial pH of 5.5 on pure glycerol as sole carbon source (n.d. – not detected). The presented data represents the mean of two biological replicates with SD≤20%.**

| **strain name** | **final pH** | **DCW [g/L]** | **Citric Acid [g/L]** | **Yield Citric Acid [g/g]** | **Mannitol [g/L]** | **Arabitol [g/L]** | **Erythritol [g/L]** | **Yield Polyols [g/g]** | **Residual Glycerol [g/L]** |
| --- | --- | --- | --- | --- | --- | --- | --- | --- | --- |
| **CBS 6124** | 1.8 | 5.0 | 22.3 | 0.24 | 3.3 | 1.8 | 0.6 | 0.06 | 5.1 |
| **CBS 7504** | 2.1 | 4.0 | 1.1 | 0.01 | 6.4 | n.d. | 1.5 | 0.10 | 23.6 |
| **DSM 1345** | 2.1 | 3.3 | 0.9 | 0.01 | 6.4 | 1.2 | 3.2 | 0.13 | 19.3 |
| **DSM 3286** | 2.1 | 4.1 | 0.6 | 0.01 | 5.2 | 1.8 | 2.9 | 0.13 | 26.5 |
| **DSM 21175** | 2.0 | 1.7 | n.d. | n.d. | 0.7 | n.d. | n.d. | 0.01 | 38.3 |
| **H222** | 2.0 | 5.8 | 1.3 | 0.01 | 14.2 | 1.3 | n.d. | 0.16 | 0.0 |
| **CBS 6114** | 2.1 | 4.2 | n.d. | n.d. | 1.7 | n.d. | 1.3 | 0.05 | 37.0 |
| **CBS 7034** | 2.1 | 3.7 | n.d. | n.d. | 2.5 | n.d. | 1.7 | 0.06 | 32.3 |
| **HA 807** | 1.9 | 5.8 | 3.0 | 0.03 | 11.0 | 1.4 | 2.3 | 0.16 | 11.0 |
| **HA 826** | 1.9 | 4.5 | 2.4 | 0.03 | 5.0 | 0.5 | 1.2 | 0.09 | 23.5 |
| **HA 827** | 2.1 | 5.0 | n.d. | n.d. | 13.1 | 2.7 | 5.7 | 0.21 | 0.0 |
| **HA 828** | 2.0 | 4.6 | 1.4 | 0.01 | 13.1 | 2.4 | 4.9 | 0.21 | 4.7 |
| **HA 829** | 2.0 | 4.6 | 1.0 | 0.01 | 14.2 | 3.5 | 7.2 | 0.25 | 0.4 |
| **HA 830** | 2.0 | 3.4 | 1.5 | 0.02 | 6.7 | 1.1 | 7.4 | 0.18 | 16.3 |
| **HA 831** | 1.9 | 5.3 | 2.1 | 0.02 | 14.2 | 2.6 | 4.9 | 0.22 | 0.9 |
| **HA 832** | 2.0 | 4.6 | 1.7 | 0.02 | 13.5 | 2.6 | 2.3 | 0.19 | 5.1 |
| **HA 833** | 2.1 | 6.8 | n.d. | n.d. | 12.3 | 0.8 | 3.2 | 0.19 | 13.4 |
| **HA 834** | 2.1 | 5.0 | n.d. | n.d. | 18.1 | 2.2 | 4.3 | 0.25 | 0.2 |
| **HA 1251** | 2.0 | 5.7 | 2.1 | 0.02 | 15.3 | 1.0 | 3.7 | 0.21 | 5.9 |
| **HA 1252** | 2.0 | 5.8 | 2.0 | 0.02 | 14.0 | 1.6 | 5.6 | 0.21 | 1.6 |
