# Supplementary material for: Metabolic Flexibility of Yarrowia lipolytica Growing on Glycerol
Source: Front Microbiol. 2017 Jan 24;8:49. doi: 10.3389/fmicb.2017.00049 (PMC5258708; doi:10.3389/fmicb.2017.00049)
Supplement: Supplementary file 2 [file Table2.DOCX]

**Table S2 – Strain screening results for twenty *Y. lipolytica* strains cultivated in bioreactors at pH 3.5 with pure glycerol as sole carbon source (n.d. – not detected). The presented data represents the mean of two biological replicates with SD≤15%.**

| **strain name** | **pH** | **DCW [g/L]** | **Citric Acid [g/L]** | **Isocitric Acid [g/L]** | **Isocitric Acid [%]** | **Yield Citric Acid + Isocitric Acid [g/g]** | **Mannitol [g/L]** | **Arabitol [g/L]** | **Erythritol [g/L]** | **Yield Polyols [g/g]** | **Residual Glycerol [g/L]** |
| --- | --- | --- | --- | --- | --- | --- | --- | --- | --- | --- | --- |
| **CBS 6124** | 3.5 | 20.7 | 26.2 | 2.7 | 9.3% | 0.29 | 9.4 | 5.4 | 1.8 | 0.16 | 0.4 |
| **CBS 7504** | 3.5 | 19.6 | 6.5 | 0.8 | 11.0% | 0.07 | 19.9 | 3.5 | n.d. | 0.19 | 1.3 |
| **DSM 1345** | 3.5 | 12.5 | 16.5 | 8.9 | 35.0% | 0.25 | 11.1 | 1.8 | 21.6 | 0.27 | 0.0 |
| **DSM 3286** | 3.5 | 14.7 | 3.0 | 0.3 | 10.4% | 0.03 | 14.8 | 6.0 | 14.1 | 0.33 | 0.0 |
| **DSM 21175** | 3.5 | 22.4 | 1.2 | 0.1 | 9.5% | 0.01 | 26.9 | 4.1 | 1.3 | 0.30 | 4.6 |
| **H222** | 3.5 | 14.9 | 19.1 | 2.7 | 12.2% | 0.22 | 18.1 | 2.3 | 6.7 | 0.16 | 0.0 |
| **CBS 6114** | 3.5 | 16.8 | 8.9 | 2.0 | 18.7% | 0.11 | 9.3 | 1.0 | 8.8 | 0.20 | 0.1 |
| **CBS 7034** | 3.5 | 23.1 | 3.2 | 0.4 | 10.5% | 0.04 | 21.9 | 5.4 | 4.7 | 0.32 | 0.3 |
| **HA 807** | 3.5 | 20.8 | 2.7 | 0.3 | 9.8% | 0.03 | 24.7 | 5.7 | 6.4 | 0.36 | 4.4 |
| **HA 826** | 3.5 | 23.5 | 3.5 | 0.1 | 1.9% | 0.04 | 24.2 | 5.9 | 4.0 | 0.35 | 3.7 |
| **HA 827** | 3.5 | 18.5 | 3.6 | 0.8 | 18.0% | 0.04 | 27.8 | 5.1 | 9.3 | 0.35 | 0.0 |
| **HA 828** | 3.5 | 22.9 | 2.0 | n.d. | n.d. | 0.02 | 24.2 | 6.7 | 9.3 | 0.42 | 3.9 |
| **HA 829** | 3.5 | 21.9 | 1.1 | n.d. | n.d. | 0.01 | 27.6 | 7.6 | 7.9 | 0.44 | 2.3 |
| **HA 830** | 3.5 | 18.8 | 2.0 | n.d. | n.d. | 0.02 | 27.7 | 5.1 | 8.7 | 0.42 | 2.3 |
| **HA 831** | 3.5 | 21.6 | 1.0 | n.d. | n.d. | 0.01 | 27.6 | 8.7 | 7.6 | 0.44 | 0.1 |
| **HA 832** | 3.5 | 23.2 | 1.6 | n.d. | n.d. | 0.02 | 28.2 | 5.9 | 3.7 | 0.38 | 0.0 |
| **HA 833** | 3.5 | 18.7 | 1.5 | n.d. | n.d. | 0.02 | 26.7 | 5.9 | 10.5 | 0.38 | 2.4 |
| **HA 834** | 3.5 | 16.4 | 2.1 | n.d. | n.d. | 0.02 | 29.7 | 7.1 | 12.5 | 0.47 | 0.0 |
| **HA 1251** | 3.5 | 20.1 | 1.5 | n.d. | n.d. | 0.02 | 30.0 | 5.6 | 6.4 | 0.30 | 4.3 |
| **HA 1252** | 3.5 | 23.4 | 1.5 | n.d. | n.d. | 0.02 | 25.2 | 5.8 | 4.6 | 0.38 | 9.2 |
