# Supplementary material for: Metabolic Flexibility of Yarrowia lipolytica Growing on Glycerol
Source: Front Microbiol. 2017 Jan 24;8:49. doi: 10.3389/fmicb.2017.00049 (PMC5258708; doi:10.3389/fmicb.2017.00049)
Supplement: Supplementary file 3 [file Table3.DOCX]

**Table S3 – Strain screening results for twenty *Y. lipolytica* strains cultivated in bioreactors at pH 5.5 with pure glycerol as sole carbon source (n.d. – not detected). The presented data represents the mean of two biological replicates with SD≤15%.**

| **strain name** | **pH** | **DCW [g/L]** | **Citric Acid [g/L]** | **Isocitric Acid [g/L]** | **Isocitric Acid [%]** | **Yield Citric Acid + Isocitric Acid [g/g]** | **Mannitol [g/L]** | **Arabitol [g/L]** | **Erythritol [g/L]** | **Yield Polyols [g/g]** | **Residual Glycerol [g/L]** |
| --- | --- | --- | --- | --- | --- | --- | --- | --- | --- | --- | --- |
| **CBS 6124** | 5.5 | 19.3 | 35.2 | 2.6 | 6.9% | 0.38 | 4.6 | 2.6 | n.d. | 0.08 | 1.5 |
| **CBS 7504** | 5.5 | 17.3 | 33.0 | 9.9 | 23.1% | 0.43 | n.d. | n.d. | n.d. | 0.00 | 0.0 |
| **DSM 1345** | 5.5 | 12.2 | 36.6 | 10.5 | 22.3% | 0.47 | 6.9 | 1.7 | n.d. | 0.09 | 0.3 |
| **DSM 3286** | 5.5 | 11.7 | 40.2 | 3.9 | 8.9% | 0.44 | 6.1 | 2.3 | n.d. | 0.09 | 0.4 |
| **DSM 21175** | 5.5 | 20.1 | 5.7 | n.d. | n.d. | 0.07 | 22.2 | 3.7 | n.d. | 0.26 | 0.2 |
| **H222** | 5.5 | 11.7 | 42.5 | 5.3 | 11.1% | 0.48 | n.d. | n.d. | n.d. | 0.00 | 0.0 |
| **CBS 6114** | 5.5 | 11.2 | 43.1 | 11.5 | 21.0% | 0.55 | n.d. | n.d. | n.d. | 0.00 | 0.0 |
| **CBS 7034** | 5.5 | 18.2 | 22.8 | 4.4 | 16.1% | 0.27 | 6.2 | 2.7 | 2.6 | 0.11 | 0.0 |
| **HA 807** | 5.5 | 24.2 | 5.8 | 3.6 | 38.6% | 0.09 | 22.4 | 5.8 | n.d. | 0.29 | 0.1 |
| **HA 826** | 5.5 | 22.1 | 6.8 | 2.2 | 24.1% | 0.09 | 22.0 | 5.9 | n.d. | 0.28 | 0.1 |
| **HA 827** | 5.5 | 18.4 | 21.4 | 4.5 | 17.3% | 0.26 | 15.1 | 2.9 | n.d. | 0.00 | 0.0 |
| **HA 828** | 5.5 | 23.6 | 2.0 | n.d. | n.d. | 0.02 | 27.6 | 6.5 | n.d. | 0.35 | 0.1 |
| **HA 829** | 5.5 | 23.7 | 2.0 | n.d. | n.d. | 0.02 | 27.2 | 7.1 | 3.6 | 0.38 | 0.1 |
| **HA 830** | 5.5 | 19.8 | 9.0 | 1.8 | 16.4% | 0.11 | 22.5 | 3.9 | n.d. | 0.26 | 0.0 |
| **HA 831** | 5.5 | 20.8 | 1.9 | 0.3 | 13.4% | 0.02 | 26.2 | 8.0 | 4.0 | 0.38 | 0.0 |
| **HA 832** | 5.5 | 21.6 | 6.1 | 1.6 | 21.0% | 0.08 | 24.6 | 5.3 | n.d. | 0.30 | 0.1 |
| **HA 833** | 5.5 | 19.9 | 5.8 | 2.4 | 29.4% | 0.08 | 14.4 | 4.4 | 11.3 | 0.30 | 0.1 |
| **HA 834** | 5.5 | 17.2 | 12.7 | 4.5 | 26.3% | 0.17 | 23.3 | 4.5 | n.d. | 0.28 | 0.0 |
| **HA 1251** | 5.5 | 19.8 | 2.4 | n.d. | n.d. | 0.03 | 33.1 | 5.0 | 2.1 | 0.40 | 0.1 |
| **HA 1252** | 5.5 | 24.0 | 4.3 | 1.6 | 26.7% | 0.06 | 28.2 | 5.9 | 1.4 | 0.36 | 0.1 |
